# Highly-sensitive wafer-scale transfer-free graphene MEMS condenser microphones

**DOI:** 10.1038/s41378-024-00656-x

**Published:** 2024-02-21

**Authors:** Roberto Pezone, Sebastian Anzinger, Gabriele Baglioni, Hutomo Suryo Wasisto, Pasqualina M. Sarro, Peter G. Steeneken, Sten Vollebregt

**Affiliations:** 1 Laboratory of Electronic Components, Technology and Materials (ECTM), Department of Microelectronics, Delft University of Technology, Delft, The Netherlands; 2 Infineon Technologies AG, Am Campeon 1-15, Neubiberg, 85579 Germany; 3 Kavli Institue of Nanoscience, Department of Quantum Nanoscience, Delft University of Technology, Delft, the Netherlands; 4 Department of Precision and Microsystems Engineering (PME), Delft University of Technology, Delft, The Netherlands

**Keywords:** Engineering, Electrical and electronic engineering

## Abstract

Since the performance of micro-electro-mechanical system (MEMS)-based microphones is approaching fundamental physical, design, and material limits, it has become challenging to improve them. Several works have demonstrated graphene’s suitability as a microphone diaphragm. The potential for achieving smaller, more sensitive, and scalable on-chip MEMS microphones is yet to be determined. To address large graphene sizes, graphene-polymer heterostructures have been proposed, but they compromise performance due to added polymer mass and stiffness. This work demonstrates the first wafer-scale integrated MEMS condenser microphones with diameters of 2*R* = 220–320 *μ*m, thickness of 7 nm multi-layer graphene, that is suspended over a back-plate with a residual gap of 5 *μ*m. The microphones are manufactured with MEMS compatible wafer-scale technologies without any transfer steps or polymer layers that are more prone to contaminate and wrinkle the graphene. Different designs, all electrically integrated are fabricated and characterized allowing us to study the effects of the introduction of a back-plate for capacitive read-out. The devices show high mechanical compliances *C*_*m*_ = 0.081–1.07 *μ*mPa^−1^ (10–100 × higher than the silicon reported in the state-of-the-art diaphragms) and pull-in voltages in the range of 2–9.5 V. In addition, to validate the proof of concept, we have electrically characterized the graphene microphone when subjected to sound actuation. An estimated sensitivity of *S*_1*kHz*_ = 24.3–321 mV Pa^−1^ for a *V*_*bias*_ = 1.5 V was determined, which is 1.9–25.5 × higher than of state-of-the-art microphone devices while having a ~9 × smaller area.

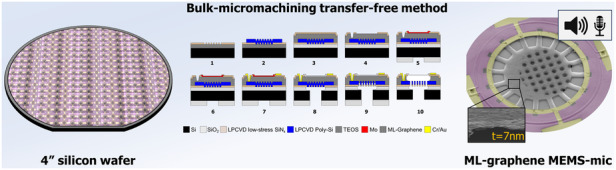

## Introduction

In the last decade, micro-electro-mechanical system (MEMS)-based microphones have become essential components for Internet of Things devices, such as smartphones or smart speakers, by supporting voice calls or control. Other application areas of MEMS microphones include voice assist, hands-free communication, and noise-cancellation. With the growing application space, future electronic consumables will contain larger numbers of MEMS microphones, allowing more complex functionalities and better acoustic interfaces. Considerable research and development efforts during the last decades have led to tremendous improvements in the MEMS microphone architecture and application-specific integrated circuit (ASIC) read-out circuits^1–3^.

From Eq. (1), to further improve microphone membrane compliance (*C*_*m*_) and device sensitivity (*S*), membrane thickness and stress need to be reduced. Both physical properties generally limit the diaphragm compliance (*C*_*m*_) and, as a result, also the device sensitivity *S*:

1 $$S=\frac{{V}_{bias}{C}_{m}}{gap}=\frac{{V}_{bias}}{gap}\frac{{R}^{2}}{4t\sigma },$$

where *V*_*bias*_ is the voltage between the microphone membrane and back-plate that are separated by a distance *gap*, *t* is the membrane thickness, and *σ* is its tensile pre-stress. To increase sensitivity, complex stress relaxation structures have been proposed to reduce the stress (*σ*)^2,4,5^. Generally, minimum thicknesses of poly-si-based membranes of *t* ≥ 450 nm are fabricated to counteract material brittleness. Eq. (1) shows that thin high-strength materials like graphene are beneficial for improving the sensitivity of MEMS microphones without additional complex architectures, because their low thickness and tension not only lead to high compliance, but also increase the resonance frequency and bandwidth of the membrane^6,7^.

Pursuing very high mechanical compliance in microphones potentially increases the output signal level. This elevated sensitivity possibly results in an improved signal-to-noise ratio (*SNR*), enabling moderate amplification by the ASIC with consequent lower noise. However, it is crucial to acknowledge that reducing the MEMS size may lead to higher viscous dissipation, resulting in amplified self-noise^8^. This highlights the need to optimize the back-plate’s design and control its viscous noise levels. Moreover, it is worth noting that increasing the signal level may come with challenges on the ASIC design concerning its output linearity (*THD*) and dynamic range, which are, however, unexplored in this paper. When dealing with high compliance and signal levels, mitigating the mechanical non-linearity of the MEMS becomes imperative.

Furthermore, one must avoid non-linearities in the ASIC output, precisely clipping with the supply voltage, to ensure a broad dynamic range and linear data acquisition even at elevated sound pressure levels. Although this work focuses mainly on improving the sensitivity and compliance of the microphone, a full benchmark of microphones also requires assessing the noise and *SNR*. For more comprehensive insights into these points, our previous work^9^ has described the *SNR* and *THD* of graphene membranes.

Numerous studies have shown graphene for this application, employing transfer methods^10–14^. These investigations have yielded multi-layer graphene membranes, often combined with polymers, exhibiting a 2*R* size of 3–5 mm. However, this contrasts with the ongoing trend toward miniaturization, seen in cutting-edge MEMS microphones which range from 0.6 to 1 mm in diameter. Also, the inclusion of polymers is often necessitated due to the microfabrication complexity and use of transfer technique, particularly in the case of involving the integration of sizeable free-standing 2D materials. These heterostructures introduce higher mass and stiffness, which can have detrimental impacts on resonance frequency and sensitivity. This study presents the first wafer-scale integration approach, merging transfer-free graphene membranes with capacitive back-plates in MEMS microphone technology. This work underscores graphene’s potential and limits against the state-of-the-art devices.

## Methods

### Bulk micromachining process flow

**Fig. 1:**
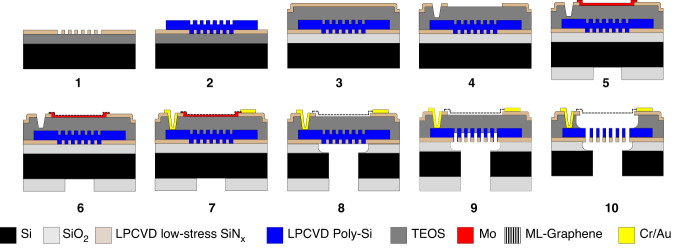
Micromachining process flow. The process steps to fabricate transfer-free multi-layer graphene condenser microphones are shown. (1) Definition of 1 *μ*m SiO_2_ landing layer and 100 nm LPCVD SiN_*x*_ etching mask for final back-side DRIE of the poly-Si venting holes. (2) LPCVD poly-Si (1 *μ*m) and patterning, (3) PECVD TEOS (5 *μ*m) and LPCVD SiN_*x*_ (100 nm), (4) Dry-etching of SiN_*x*_ for membrane area definition and vias for bottom-electrode contacts. (5) Mo sputtering (50 nm) and patterning, (6) CVD run for graphene growth, (7) Cr/Au 20/200 nm evaporation and lift-off, (8) Bosch process and SiO_2_ removal, (9) DRIE of poly-Si and (10) VHF of sacrificial layer

A 100 mm p-type silicon wafer is thermally oxidized at 1000 °C forming 1 *μ*m SiO_2_ film as an insulating layer from the back-plate and as landing layer for final bulk silicon etching. A layer of 100 nm Low-Pressure Chemical Vapor Deposition (LPCVD) SiN_*x*_ (SiH_2_Cl_2_295 sccm/NH_3_105 sccm) is deposited at 850 °C and patterned in correspondence of the future venting holes of the suspended back-plate (Fig. 1(Step 1)). LPCVD is also used to deposit 1 *μ*m of poly-Si (SiH_4_ 45 sccm) at 605 °C with consequent Boron doping with 45 keV, and 10^15^at/cm^2^. After an annealing doping activation step of 1 h at 950 °C in N_2_/Ar atmosphere, the continuous poly-Si layer is patterned to define the back-plate area with Cl/HBr chemistry (Fig. 1(Step 2)). Then, as a future sacrificial layer, a Plasma-enhanced chemical vapor deposition (PECVD) Tetraethyl orthosilicate (TEOS) film of 5 *μ*m is deposited and annealed at 1000 °C in Ar/N_2_ environment. A second film, adopted as capping and clamping area for the final sacrificial etching of LPCVD SiN_*x*_ (100 nm) is deposited (Fig. 1(Step 3)) and etched accordingly to the future graphene suspended area and vias for the counter electrode contacts (Fig. 1(Step 4)). A thin film of 50 nm Mo is sputtered at low temperature 50 °C and etched by dry-etching with Cl/O_2_ chemistry (Fig. 1(Step 5)). Graphene is then synthesized at 935 °C with an in-house reactor 4-inch AIXTRON “Black Magic Pro" in a pressure 25 mbar with H_2_ as a reducing agent of oxidized Mo, and a CH_4_ step for the growth (Fig. 1(Step 6)). More details about the involved reactor can be found in previous Jan Mischke et al. work^15^. Next, Cr/Au (20/200 nm) are evaporated by ion-beam evaporation in a vacuum and patterned using a lift-off technique with acetone at 40 °C, IPA, and DI-water (Fig. 1(Step 7)). Bosch cavity etching is performed on the backside of the 100 mm wafer, and the SiO_2_ (1 *μ*m) is wet-etched in Buffered oxide etch (BOE) 6:1 chemistry (Fig. 1(Step 8)). A final deep reactive ion etching (DRIE) through the back-side is performed to completely etch the exposed poly-Si in correspondence with venting holes using the SiN_*x*_ layer as an etching mask (Fig. 1(Step 9)). Mo is finally etched with H_2_O_2_ and gently washed with DEMI-water to remove all etching by-products. After dicing of 1 cm × 1 cm chips where several devices are included, the vapor hydrofluoric acid (VHF) etch is performed at 45 °C with 100% anhydrous HF, N_2_, EtOH in a commercially available Primaxx *μ*Etch system at 125 Torr (Fig. 1(Step 10)).

## Results and discussion

### Design concept and fabrication

**Fig. 2:**
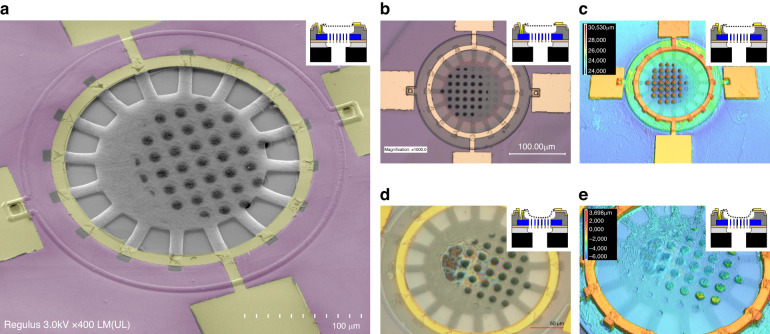
Device visualization by SEM and 3D laser scanning confocal microscope. **a** SEM false color image of one final device (geom. C) in tilted view and low-magnification mode. Partial Cr/Au cracks are present on top of the multi-layer graphene tethers due to thermal stress experienced during the Cr/Au evaporation. A slower evaporation rate is found to improve the reported state. Undesired mask shift during the back-alignment step in hard contact resulted in misalignment and the unintended closure of venting holes. **b** Optical microscope image of the same device as in (**a**). **c** Laser topography image of the same device as in (**a**). **d**, **e** Optical microscope, and topography images of a collapsed device. As inset in (**c**, **e**), a height scale is added showing a downward deformation of the poly-Si back-plate due to thermal stress of ≈1.5% (center) of the suspended region. The main source of the compressive stress can be a residual thin layer of TEOS

We characterize MEMS microphones with three main graphene membrane geometries: trampoline membranes (Fig. 2) with 2*R* = 320 *μ*m (geom. A), and 220 *μ*m (geom. C) and a fully clamped one with 220 *μ*m (geom. B), as accurately described in Fig. S1 (Supplementary material). The sacrificial layer gap of 5 *μ*m is chosen for all device geometries since they are all processed in the same 100 mm wafer. Although a thinner sacrificial layer might increase readout sensitivity, it has not initially been planned because it might increase damping effects and decrease the yield of the final membrane release. Also, decreasing the distance between the plates, to target high aspect ratios ($\frac{2R}{gap}$) translates into very high fabrication complexity and reliability due to hydrogen bridging, capillary, electrostatic, and Van Der Waals forces that lead to membrane collapse^16^. For such types of highly compliant membranes, larger gaps are also preferred to keep a great dynamic range. The trampoline designs are observed to result in a higher yield (70% for >20 devices) than the fully clamped designs (18% for >80 devices), which we attribute to the reduced capillary forces as a consequence of the lower liquid volume and larger etching window. The yield of the fabricated membranes is also found to be affected by the electrode areas that cover the graphene edges at the clamping region (Fig. S2, Supplementary material).

The selection of a back-plate thickness of *t* = 1 *μ*m is aimed at mitigating self-noise arising from the oscillatory air movement within the elongated openings of the back-plate, as discussed in the work by S. Shubham et al^5^. Earlier research on graphene microphones employed counter electrodes with thicknesses exceeding *t* > 100 *μ*m^10,11,13,14^. It is noteworthy that none of these prior works embraced a wafer-scale fabrication methodology, which holds potential for seamless integration with ASICs and facilitating large-scale manufacturing processes.

After finalizing the devices, they are visually inspected in Fig. 2b–d to assess the fabrication process. Using the topography mode in a Keyence VK-X250 confocal microscope we determine which microphone membranes are suspended. In Fig. 2d, the measured height in the venting holes corresponds to the case of a collapsed device, while Fig. 2c shows a suspended one. The membrane thickness of *t* < 10 nm is measured with an atomic force microscope (AFM) from Cypher Asylum Research in air topography mode to determine the multi-layer graphene thickness (*t*) following the procedure described in Fig. S3 (Supplementary material), and previous work^17^. An AFM image of the proposed multi-layer graphene before the VHF step is presented in Fig. S3d (Supplementary material). Some polymer residuals on the membrane are also found due to the lift-off step. However, they can easily be avoided with an encapsulation layer such as ALD AlO_*x*_^18^ which would also be compatible with the proposed process flow.

**Fig. 3:**
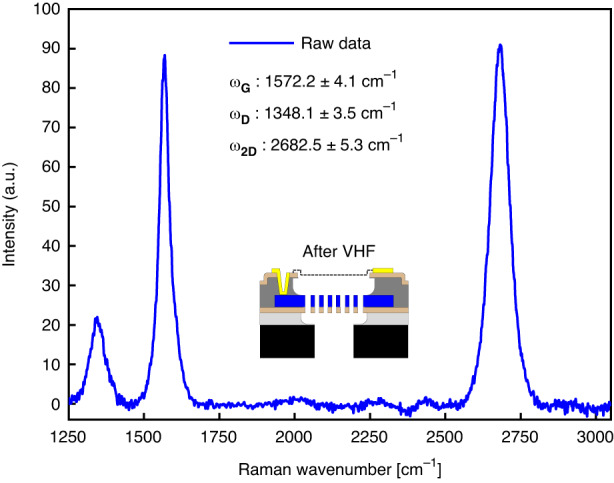
Raman spectroscopy. An example of material crystallinity at the end of the process after final release is shown in a wavenumber range of 1250–3000 cm^−1^. As an insight, all mean values and standard deviations are summarized. All acquisitions have been performed on the suspended multi-layer graphene in correspondence with the venting holes. Taking point in the free-standing area in correspondence with the back-plate leads to incorrect measurements due to suspended polysilicon influence on the backscattered signal

A Horiba HR800 Raman spectrometer equipped with a 514.4 nm Ar+ laser, ×100 objective with a NA of 0.9 is used for the crystallinity characterization of the multi-layer graphene. In Fig. 3, a Raman spectrum of a suspended microphone membrane, after finalizing the complete process is presented. The peak position values provided as an inset in the graph are based on three inspected membranes where different locations have been averaged three times within the same acquisition. All the data are fitted with Lorentzian functions to determine the crystallinity imprint of the proposed material. The *ω*_*D*_, *ω*_*G*_, *ω*_2*D*_ are centered in 1348.1 cm^−1^, 1572.2 cm^−1^, and 2682.5 cm^−1^ with standard deviations of 4.1 cm^−1^, 3.5 cm^−1^, and 5.3 cm^−1^. The full-width half-maximum FWHM at the points D, G, and 2D are calculated from the Lorentzian fits as 58.3, 47.3 and 76.9 cm^−1^. In addition, the *I*_*D*_/*I*_*G*_ and *I*_2*D*_/*I*_*G*_ are found to be 0.2 ± 0.03, 0.89 ± 0.25. These measurements are typical for multi-layer Mo-grown graphene, where based on the FWHM 2D, it can be characterized as turbostratic graphene, where the stacked layers are more twisted oriented^19^. Furthermore, we have not found evidence of damage due to final DRIE and VHF etching, as also shown in previous work^17^. We conclude that the Raman data indicate the low invasiveness of the presented process flow since the defectivity, as obtained from the Raman peak positions, is similar to other work based on the same material^20–22^.

### Eigenfrequency analysis

**Fig. 4:**
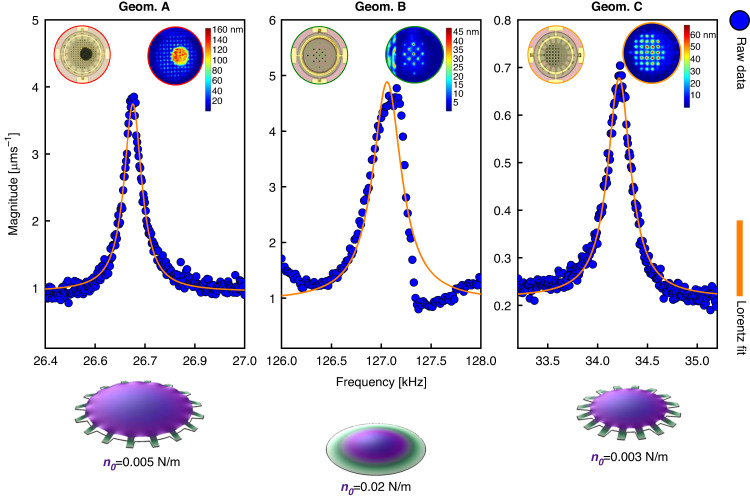
Membrane eigenfrequency. First resonance frequency modes of the three different membranes are compared and fitted with Lorentz functions. The insets show stroboscopic topography data of the +z membrane displacement through the venting holes. Below, is a Comsol simulation of the mode-shapes of each of the geometries for different pre-tensions to match the experimental resonance frequencies

Concerning the potential of these devices as microphones, the fundamental resonance frequency *f*_01_ is of central importance. A resonance frequency *f*_01_ that is within the audible frequency range can negatively affect the flatness of the microphone response and sound recording. To assess the resonance frequencies, all geometries are investigated, and the three respective measurements are plotted in Fig. 4. Digital holographic Lyncée Tec DHM R2200 microscopy is used to visualize and quantify the first mode shape (*f*_01_) in stroboscopic mode. These membranes show resonance frequencies above the audible range (*f*_01_ > 20 kHz) at 1 × 10^−3^ mbar by piezo-shaker actuation. With the amplitude and phase acquisition by holographic microscope LynceeTec and Koala Software, the mode shapes are formed after post-processing with the MEMS Analysis Tool (Vibration Maps), proving that we deal with the fundamental modes. A visual example of the dynamic motion with the upward and downward membrane displacement while imaging through the venting holes is shown in Movie 1 (Supplementary material). The utilization of venting holes for visualization serves to mitigate amplitude errors arising from the less transparent polysilicon area. This approach is crucial, as the height profile in correspondence to the polysilicon is inaccurately captured. Energy losses and dampening are minimized due to the low pressure of 1 × 10^−3^ mbar. In addition, the study incorporates a Polytec MSA-400 operating in scanning mode by piezo-shaker actuation to assess membrane velocities at resonance frequencies, where membrane motions are observed. This analysis involves again the piezoelectric actuation at low-pressure conditions (Fig. 4).

Considering an undamped circular drum vibrating in its linear regime, the physical parameters associated with the solution of a harmonic oscillator, can be extracted from Eq. (2)^6^. For the fundamental mode, the *k*_01_ = 4.8967 *n*_0_ is the modal stiffness, *n*_0_ is the pre-tension, the *m*_01_ = 0.2695 *m* is the modal mass, *m* = *ρhπR*^2^ is the total mass, *ρ* is the mass density of the graphene, and *t* is the membrane thickness:

2 $${f}_{01}=\frac{1}{2\pi }\sqrt{\frac{{k}_{01}}{{m}_{01}}}=\frac{2.405}{2\pi R}\sqrt{\frac{{n}_{0}}{\rho t}}$$

Considering *t* = 7 nm, *ρ* = 2267 kg/m^3^, and *R* = 110, 160 *μ*m, without involving any possible polymer residuals or wrinkle influences, the extracted pre-tension from Eq. (2) for the three geometries are 0.002 N/m (geom. A), 0.02 N/m (geom. B) and 0.0015 N/m (geom. C). Furthermore, based on the equation that relates pre-stress *σ* to pre-tension *n*_0_: *σ* = *n*_0_*t*, the calculated residual stresses are 0.28 MPa (geom. A), 2.8 MPa (geom. B) and 0.21 MPa (geom. C).

The three geometries are also modeled with Finite Element Analysis (FEA) to match the experimental eigenmodes. The FEA results are successfully obtained for the *n*_0_ described in Fig. 4. Differently than the fully clamped geometry (geom. B) where the analytical *n*_0_ = 0.02 N/m is equal to the FEA results, geom. A and C show different values of *n*_0_ = 0.005 N/m (*σ* = 0.71 MPa) and 0.003 N/m (*σ* = 0.42 MPa) compared to the analytical results. These differences with Eq. (2) (valid for fully clamped) are attributed to differences between a circular drum and a trampoline.

### Base capacitance and pull-in

**Fig. 5:**
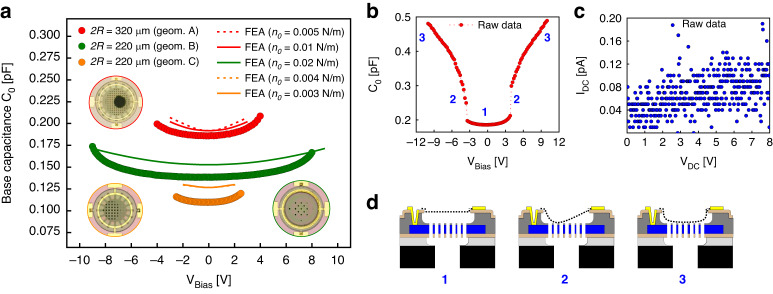
Base capacitance and pull-in. **a** *C*_0_ – *V*_*bias*_ curves of the three geometries are compared with FEA results. The devices are driven with *V*_*AC*_ = 100 mV and *f*_1_ = 100 kHz. **b** *C*_0_ – *V*_*bias*_ linear sweep from −9.5 V to 9.5 V describes the asymmetric membrane displacement of geom. **b**, **d** The blue numbers describe the membrane dynamics under *V*_*bias*_ increase of the inspected Geom. A device (also in Movie 2, Supp. mat. for geom. C). **c** Both electrodes are driven with a *V*_*bias*_ linear sweep from 0 V to 8 V. Despite the partial membrane collapse at *V*_*bias*_ = 3.8 V, no short circuits are found due to a residual TEOS thin layer (geom. A). **d** Membrane deflection under non-uniform electrostatic forces

Defining the operational voltage window is also fundamental to validate the suitability of the proposed devices for this microphone application. The functionality of a condenser microphone exists for *V*_*bias*_ < *V*_*pull*−*in*_, since for higher voltages, the membrane snaps on the back-plate electrode. In Fig. 5a, capacitive-voltage *C*_0_ − *V*_*bias*_ curves indicate the voltage window of the fabricated devices compared with FEA simulations. These electrical measurements are performed with a Cascade Summit probe station connected to an Agilent 4294A Precision Impedance Analyzer. With *V*_*bias*_ increase, the base capacitance *C*_0_ (Eq. (3)), increases with a non-linear trend. This is explained by the gradient of energy generated by the electrostatic forces that scale quadratically with voltage and decrease the gap between the membrane and the back-plate. The three geometries (geom. A, B, C) show pull-in at a voltage *V*_*pull*−*in*_ in a range of 2.0–9.5 V based on ten inspected devices:

3 $${C}_{0}=\frac{{\epsilon }_{0}{\epsilon }_{medium}A}{gap}$$

The experimental *C*_0_ and *V*_*pull*−*in*_ results are compared to FEA results of the same active free-suspended area, with the extracted pre-tension *n*_0_ shown in Fig. 4. Parasitic capacitances (*C*_*p*_), which correspond to the common area between the electrodes and the back-plate, are analytically calculated using the parallel plate approximation and added to the FEA results. The experimental capacitances *C*_0_ are similar to the FEA with an error bar of <15%, which might be addressed to device imperfections like film deformations (stress-induced) of the counter electrode. The minimum capacitance does not occur exactly at *V*_*bias*_ = 0*V*. From a parabolic fit of the experimental *C*_0_–*V*_*bias*_ in Fig. 5a, the three geometries show built-in voltages *V*_*bi*_ = − 316, −289 and −219 mV. The reason might arise from a poor connection between the metal and the graphene or Si, or it could be associated with residual trapped charges in the TEOS.

The fully clamped geometry shows the highest *V*_*pull*−*in*_ ≈ 8.5 V, which corresponds to a static displacement of approximately 1.65 *μ*m, that is ≈1/3 of the gap based on FEA analysis in Fig. S4 (Supplementary material). This is expected as this device also showed a higher resonance frequency and stiffness accordingly with the previously reported *f*_01_ due to the higher stiffness in Fig. 4. It is shown in Fig. 5b, that above *V*_*pull*−*in*_ the capacitance *C*_0_ continues to increase, possibly due to an increase of the contact area with increasing voltage (see Fig. 5d and Movie 2 Supplementary material).

As follows, increasing *V*_*bias*_ > *V*_*pull*−*in*_, *C*_0_ shows a different trend that is not based on a parabola-shaped deflection, which is usually assumed for circular plates. The exact membrane collapse is summarized in three steps in Fig. 5d and Movie 2 (Supplementary material). Also, all inspected membranes are found to restore to their original state after the entire collapse for *V*_*bias*_ = *V*_*pull*−*out*_ ≤ 15 − 30 % of *V*_*pull*−*in*_, showing the typical hysteresis behavior originating the electrostatic force non-linearity. An example of the entire hysteresis cycle is shown in Fig. S5 (Supplementary material). In Fig. 5c, the *V*–*I* curve shows no significant electrical leakage current between the top and bottom electrodes (*I*_*leakage*_ < 0.20 pA). Current leakage is possibly limited by the presence of a thin residual layer of unetched TEOS on top of the back-plate.

### Device response under sound actuation

In this study, the three geometries (Geom. A, B, and C) are subjected to sound pressure excitation at a constant *p* = 1 Pa, following proper calibration within the frequency range of 10–10,000 Hz. Laser Doppler Vibrometry (LDV) is employed to capture the corresponding membrane motions as described in Fig. S6 (Supplementary material). Notably, all responses displayed a low-pass trend without any indications of Low-Frequency Roll-Off (LFRO). The mechanical compliances for Geom. A, B, and C were found to be 1.07, 0.081, and 0.56 *μ*m, respectively, when subjected to 1 kHz and 1 Pa actuation. These values align closely with findings from prior research on the same free-standing graphene^9,17^, highlighting remarkably high mechanical compliances without the counter electrode or capacitive architectures. Notably, comparable high compliances for small diameters (2R < 320 *μ*m) have not been reported in other existing literature.

Displacement variations are further found in relation to distinct membrane diameters and pre-tension values, obtained through FEA as illustrated in Fig. 4. By utilizing the damped harmonic oscillator represented by Eq. (4), the experimental results are compared with the analytical response, resulting in a fit with the pre-tension (*n*_0_) extracted from FEA eigenfrequency, exhibiting an error range of 6-25 % and the respective geometry radius (*R*):

4 $${C}_{m}(\omega )=\frac{{R}^{2}}{4{n}_{0}\sqrt{{(1-\frac{{\omega }^{2}}{{\omega }_{0}^{2}})}^{2}+\frac{{\omega }^{2}}{{\omega }_{0}^{2}{Q}^{2}}}}$$

The observed low-pass behavior is predominantly attributed to the back-plate design, which introduced acoustic resistance. This is primarily due to the presence of air volume within the perforations and the consequent impact of the air mass (inertia) on its movement through the holes, leading to damping effects caused by membrane displacement. A comprehensive analysis of damping for the suggested devices is further elaborated in Section S7 (Supplementary material). Lumped-element model circuits are employed to replicate the device responses across various damping scenarios. To enhance the system response with a flatter broader bandwidth, it is advisable to increase the gap between the membrane and back-plate, or maximize the size of the perforations while reducing their pitch with a consequent sensitivity reduction accordingly with Eq. (1). Another potential cause for the relatively high motion of the membrane at low frequencies could be wind noise actuation^23^.

The damping effect caused by the back-plate on the frequency response of MEMS condenser microphones has been already explored on silicon-based diaphragms showing similar trends^24,25^.

**Fig. 6:**
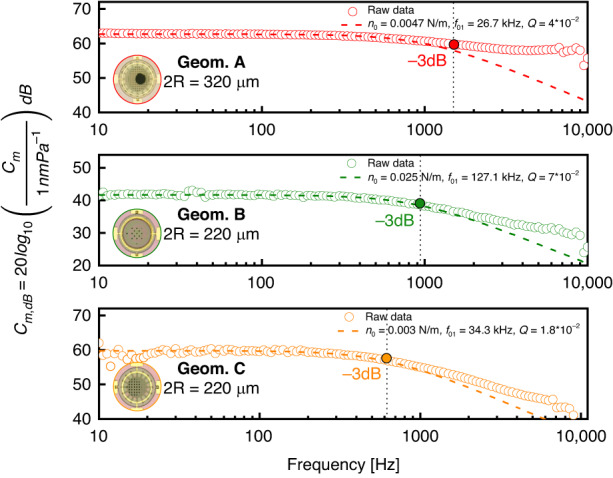
Mechanical sensitivity at 1Pa in 10Hz - 10kHz. In the legends are described the main parameters that have been used to fit the data with (Eq. (4)). The slope response for *f* > 10 Hz is mainly affected by *n*_0_ and the quality factor (*Q*). A better fitting for all proposed geometries in the slope for *f* > 800 Hz is found for low *Q* (overdamped systems) and original natural modes *f*_01_ values. The cut-off frequencies are 1500 Hz (Geom. A), 940 Hz (Geom. B), and 620 Hz (Geom. C)

Despite the low-pass behavior shown in Fig. 6, the measured devices still demonstrate substantial compliance as they approach 10 kHz, with corresponding amplitudes of 794, 31, and 19 nm for geometries A, B, and C, respectively. These values exceed the mechanical compliances typically reported in the literature for silicon-based diaphragms, which usually feature membrane diameters 3-4 × greater than the results shown in this study^9^. Further improvements to the flatness of the response of the microphones will require more engineering efforts or might be mitigated using signal processing techniques to correct for the frequency response.

The measurements are limited to 10 kHz due to the presence of a sealed chamber utilized for monitoring pressure changes via a reference microphone. This limitation arises from the Helmholtz resonance observed around this frequency, stemming from the volume discrepancy between the speaker area (inside the chamber) and a larger opening where the sample is securely affixed, ensuring minimal pressure leakage, and thus assuring precisely a difference in pressure of 1 Pa. This arrangement allows the devices to receive sound waves from the back-plate as in Fig. S6 (Supplementary material).

**Fig. 7:**
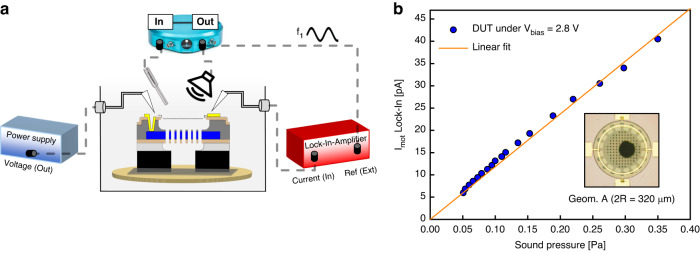
Electrical response under sound actuation. **a** The measurement setup employed for characterizing the device under test involves several components. The sample is secured to the chuck using double-sided tape covering the entire back side of the chip. Practical constraints drive this decision, as vacuum fixation is unfeasible within the experimental setup. Double-sided carbon tape serves the dual purpose of ensuring chip stability and ensuring that the sound pressure waves are only incident on the top side of the microphone. We note that this results in a relatively small back volume that increases the effective stiffness of the microphone compared to the configuration in Fig. 6. To bias the device, a power supply is utilized, which connects to the counter electrode. Simultaneously, a *V*_*AC*_ driving signal from Moku:Lab (output) is employed to drive the speaker and serves as the external reference signal for the lock-in amplifier SR830. The membrane electrode is connected to the input of the lock-in amplifier in current mode. The LabVIEW script records the current output obtained from the lock-in amplifier. Additionally, the sound pressure is captured using a reference microphone connected to the Moku:Lab (input) positioned in close proximity to the device under test. **b** A trampoline with geom. A is driven by sound at *f*_1_ = 3 kHz at different pressure sound waves amplitudes *p* = 0.05–0.35 Pa. The *I*–*p* experimental curve is fitted with a linear fit

Finally, we characterize the electrical readout of a device under test (DUT) of Geom. A with *V*_*pull*−*in*_ = 3.8 V. The device is actuated by sound pressure with respective pressure amplitudes of *p* = 0.05–0.35 Pa. To detect the electrical response from the DUT, the counter electrode is biased with a *V*_*bias*_ = 2.8 V with a BK Precision 9130 DC Power Supply, and the capacitive current that flows via the ML-Gr membrane is to monitor for the membrane motion as shown in Fig. 7a. In this scenario, synchronization and data acquisition involve the utilization of a LabVIEW script to control a Stanford SR830 Lock-In Amplifier (LIA). The AC current from the DUT is acquired using this setup. Additionally, the reference microphone signal is received through a MOKU:Lab operating in IN/OUT mode, enabling the calculation of sound pressure. Furthermore, the MOKU:Lab also provides an AC voltage signal to drive the speaker and serves as the signal reference for the lock-in detection. The measurements in Fig. 7 involve a high mid-range frequency *f*_1_ = 3 kHz determined by the emitted sound frequency of a reference micro-speaker used in this specific setup. Considering the constraints of the setup, a small speaker is used, which cannot generate high sound pressures at lower frequencies.

Under sound pressure, the induced motion generates a current due to the time-dependent charge variation ($\frac{{{\Delta }}Q}{{{\Delta }}t}=\frac{{{\Delta }}(CV)}{{{\Delta }}t}$) of the proposed capacitor. Thus, by increasing the sound pressure, the distance between the electrodes reduces forcing higher motion current *i*_*mot*_ as in Eq. (5):

5 $${i}_{mot}(t)={V}_{bias}\frac{{{{\rm{d}}}}}{{{{\rm{d}}}}t}\int\nolimits_{0}^{R}\frac{2\pi rdr}{gap-{x}_{sound}{(1-\frac{{r}^{2}}{{R}^{2}})}^{2}}\approx \frac{{V}_{bias}\pi {\epsilon }_{0}{R}^{2}}{3ga{p}^{2}}\frac{{{{\rm{d}}}}}{{{{\rm{d}}}}t}x(t)$$

The equation deals with determined capacitance in the context of a fully clamped circular membrane, assuming that the deflection (*x*_*sound*_) remains smaller than the gap between both electrodes. Thus, the sound pressure magnitude is proportional to the membrane displacement amplitude *x*_*sound*_. In this way, the amplitude of the graphene displacement under sound actuation *x*_*sound*_ can be extracted from the following equation Eq. (6):

6 $$| {x}_{sound}| =\frac{3ga{p}^{2}}{{V}_{bias}{\epsilon }_{0}A\omega }| {i}_{mot}(t)|$$

In Fig. 7b, the relationship between the current output of the LIA and sound pressure is depicted, and it exhibits a trend that is compared to a linear fit. Specifically, the current from the LIA represents the amplitude of the induced current at the driving sound frequency. By utilizing Eq. (6), considering the highest device response under *p* = 0.35 Pa of *i*_*mot*_ = 40 pA, an estimated membrane displacement of *x*_*sound*_ ≈ 141 nm is calculated. Upon normalization of the measured value at 0.35 to 1 Pa, a displacement of *x*_*sound*_ ≈ 403 nm is obtained.

Remarkably, this value differs by approximately 2.6 × from the experimental results depicted in Fig. 6. The observed amplitude difference is expected to be influenced by the *V*_*bias*_ effect on the membrane dynamics. It is possible that the *V*_*bias*_ causes a hardening effect, potentially leading to the elimination of foldings or wrinkles, thereby stretching the membrane and increasing its stiffness. Also, the observed stiffening of the diaphragm might result from the electrostatic force causing a nonlinear static displacement in the diaphragm^5^. These factors could contribute to a significant effect on the final device response. Unlike the mechanical compliance measurements, at this time, the cavity can also exhibit a form of spring-like behavior, wherein when the membrane is deflected, the enclosed air volume undergoes compression and expansion. This phenomenon contributes to the generation of a counteractive force, effectively reducing the membrane’s compliance. In the end, a current attenuation can also be attributed to the current leakage through the wiring connections. However, to gain a deeper understanding and establish a more comprehensive understanding of the influence of the *V*_*bias*_ on mechanical compliance, further experiments are required, thereby paving the way for future research in 2D materials integration for MEMS condenser microphones.

## Conclusion

This research presents a route for integrating multi-layer graphene into condenser MEMS microphones without the need for transfer or polymer support. This novel approach effectively addressed previous limitations associated with fabricating graphene microphones on a wafer scale without polymer supports. Several designs using this method, enabling the tuning of device sensitivity, are proposed. Notably, the resulting devices exhibited *V*_*pull*−*in*_ = 2–9.5 V, making them compatible for future ASIC integration. Despite the limited acoustic bandwidth, attributed primarily to the counter electrode design, the devices demonstrated an estimated sensitivity of up to *S*_1*kHz*_ = 24.3–321 mV Pa^−1^. This sensitivity is more than two times of the reported state-of-the-art MEMS microphones, despite having a diameter 3 × smaller. While the proposed devices exhibit very high sensitivity, further exploration and enhancement of many more relevant performance metrics of microphones need to be considered in future work. This will be critical before graphene microphones can outperform commercial devices in all aspects. Finally, this study unveiled a promising and viable way for integrating multi-layer graphene into condenser MEMS microphones opening up new possibilities for future microphone technology.

### Supplementary information

Supplementary material

Movie 1: LyncéeTec membrane modes (Geom.C)

Movie 2: Pull-in, and pull-out recordings (Geom.C)
