# Correction: Highly-sensitive wafer-scale transfer-free graphene MEMS condenser microphones

**DOI:** 10.1038/s41378-024-00705-5

**Published:** 2024-06-04

**Authors:** Roberto Pezone, Sebastian Anzinger, Gabriele Baglioni, Hutomo Suryo Wasisto, Pasqualina M. Sarro, Peter G. Steeneken, Sten Vollebregt

**Affiliations:** 1 Laboratory of Electronic Components, Technology and Materials (ECTM), Department of Microelectronics, Delft University of Technology, Delft, The Netherlands; 2 Infineon Technologies AG, Am Campeon 1-15, Neubiberg, 85579 Germany; 3 Kavli Institue of Nanoscience, Department of Quantum Nanoscience, Delft University of Technology, Delft, the Netherlands; 4 Department of Precision and Microsystems Engineering (PME), Delft University of Technology, Delft, The Netherlands

**Keywords:** Engineering, Electrical and electronic engineering

Correction to: *Microsystems & Nanoengineering*

10.1038/s41378-024-00656-x published online 21 February 2024

After publication of this article^1^, it was brought to our attention that ***two pressure values*** were not correctly copied from the submitted original work to the published version.

**Correction 1 (from PDF, Page 4 of 9)**: “These membranes show resonance frequencies above the audible range (*f*_01_ > 20 kHz) at 1 × **10**^**3**^ mbar by piezo-shaker actuation”. The described phrase needs to be changed reporting the right pressure value of 1 × **10**^**−3**^ mbar. The new phrase will be: **“*These membranes show resonance frequencies above the audible range (f***_***01***_ ***>*** ***20*** ***kHz) at 1*** × ***10***^**−*3***^ ***mbar by piezo-shaker actuation*”**.

**Correction 2 (from PDF, Page 4 of 9)**: “Energy losses and dampening are minimized due to the low pressure of 1 × **10**^**3**^ mbar”. Again, the described phrase needs to be changed reporting the right pressure value of 1 × **10**^**−3**^ mbar. The new phrase will be: **“*Energy losses and dampening are minimized due to the low pressure of 1*** × ***10***^**−*3***^ ***mbar*”***.*
