# Supplementary material for: Highly-sensitive wafer-scale transfer-free graphene MEMS condenser microphones
Source: Microsyst Nanoeng. 2024 Feb 21;10:27. doi: 10.1038/s41378-024-00656-x (PMC10879197; doi:10.1038/s41378-024-00656-x)
Supplement: Supplementary file 1 — Supplementary material [file 41378_2024_656_MOESM1_ESM.pdf]

Supplementary material: Highly-sensitive wafer-scale transfer-free graphene MEMS condenser microphones

Roberto Pezone,[*,†] Sebastian Anzinger,[‡] Gabriele Baglioni,[¶] Hutomo Suryo Wasisto,[‡] Pasqualina M. Sarro,[†] Peter G. Steeneken,[§,¶] and Sten Vollebregt[†]

†*Laboratory of Electronic Components, Technology and Materials (ECTM), Department of Microelectronics, Delft University of Technology, The Netherlands*

‡*Infineon Technologies AG, Am Campeon 1-15, Neubiberg 85579, Germany*

¶*Kavli Institue of Nanoscience, Department of Quantum Nanoscience, Delft University of Technology, the Netherlands*

§*Department of Precision and Microsystems Engineering (PME), Delft University of Technology, The Netherlands*

E-mail: r.pezone@tudelft.nl,s.vollebregt@tudelft.nl

Phone: +31 152789437

S1. Design concept

The main geometry parameters of the proposed devices are shown in Figure S1. These refer to the ideal case where no residual etching of the etching mask SiN_x, under etching, and deformations by stress are considered for simplicity. The design variables are related to the clamping geometry and the free-standing diameter and the air cavity. In geom. A, the black hole is a result of long over-etching under isotropic SF_6 that has merged several venting holes resulting in a fully etched region.

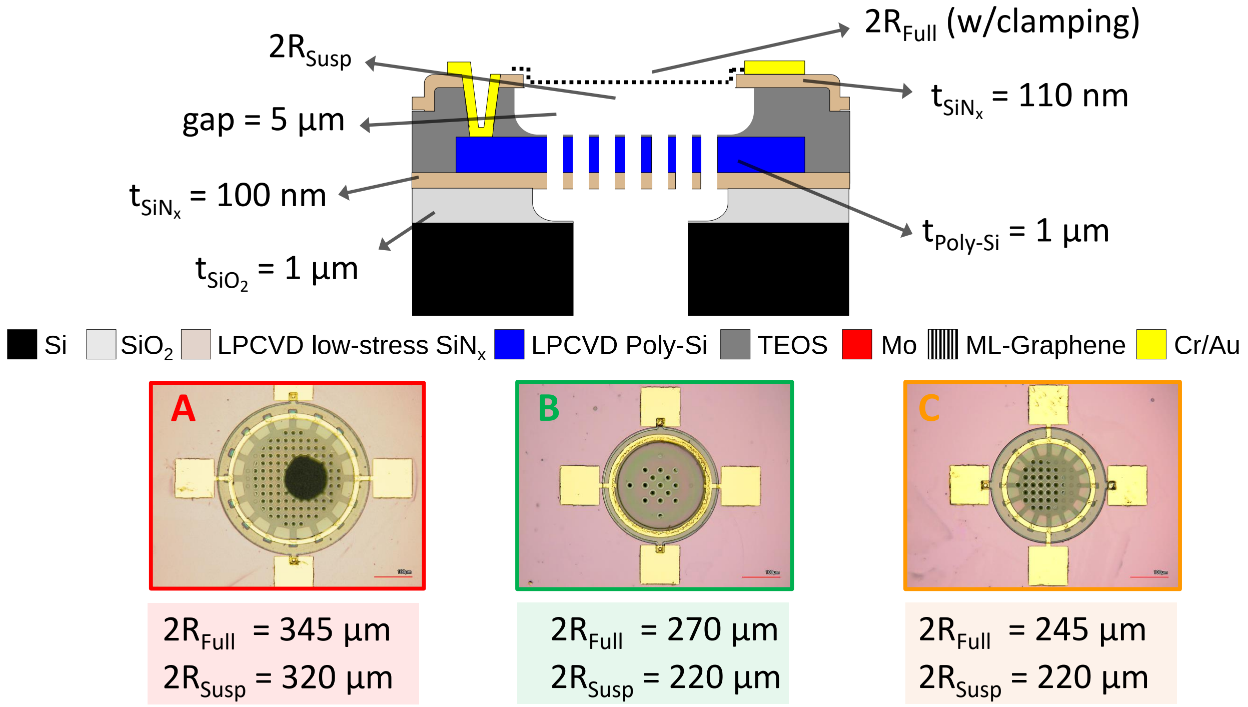

Figure S1: **Geometry schematic of the proposed devices.** Optical microscope images of devices (after VHF) of the three geometries are shown.

S2. VHF and clamping geometry

During the isotropic VHF, the top layer etching mask SiN_x is partially etched, and in correspondence of graphene edges, where no Cr/Au is present, graphene starts to delaminate with higher chance (Figure S2). A SEM image of a broken device is shown to highlight the over-etched Polysilicon back-plate as discussed in Figure S1. Membrane cuts can be originated due to large folding after delaminations or residual scratched regions due to wafer handling of the final DRIE step where the front-side faces the mechanical arm and chuck. A possible solution would be an extra capping material such as ALD thin films or polymers.

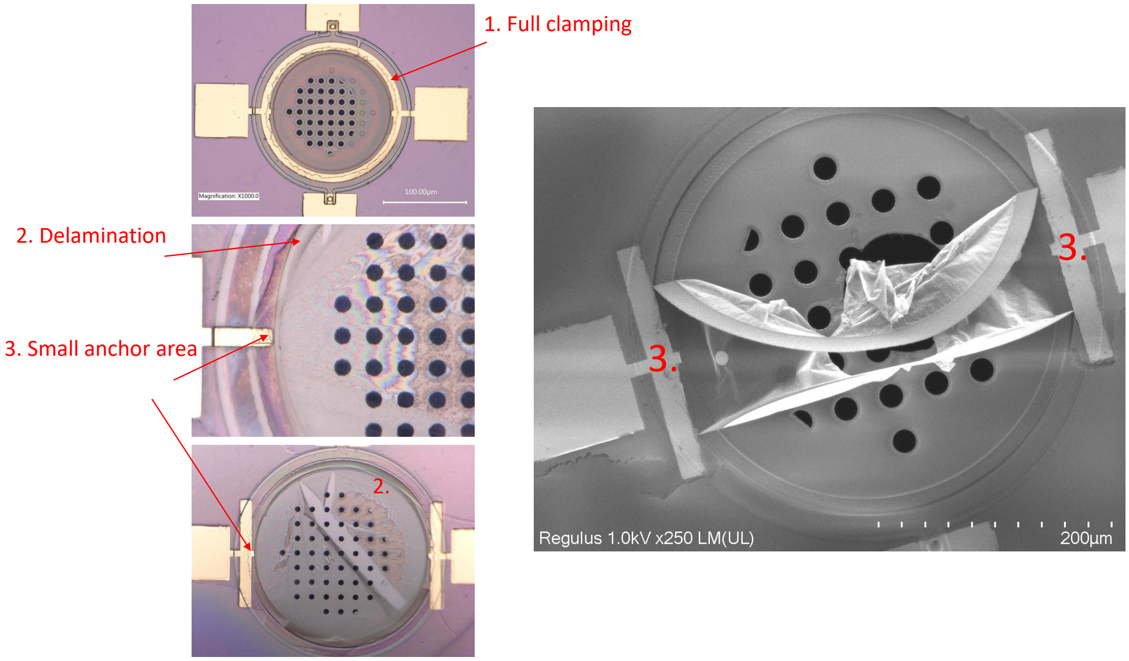

Figure S2: **Comparison of over-etched and broken devices.** An image comparison of a suspended device (geom. B), partially suspended, and broken devices. It is evident the effect of a full electrode clamping that helps to avoid larger delaminations.

S3. Thickness measurement

The AFM thickness measurements are obtained on graphene which is processed with all the reported steps except the VHF. With mechanical shaking and high surface tension of the DI-water, the graphene delaminates and it is scooped on a flat thermal SiO_2 (Figure S3c).

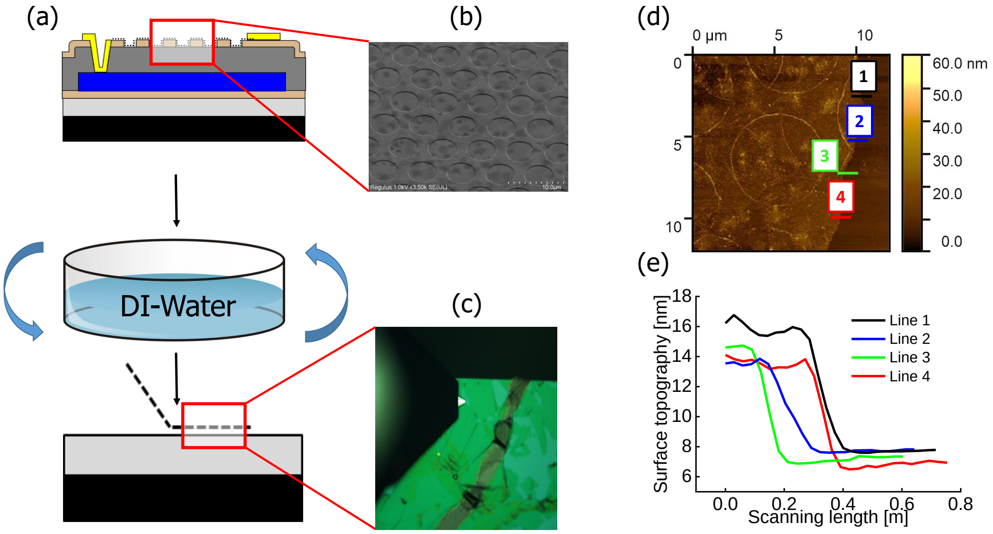

Figure S3: **Graphene thickness.** (a) Wet-transfer method is used to transfer a part of 5 mm x 5 mm patterned area of the graphene, designed and fabricated in the same mask-set for other application. (b) SEM image of the patterned region in a tilted view where graphene is conformally covering the entire topography. (c) Optical image through the AFM camera of the inspected area. Large foldings are present due to the rough transfer step. The flattest areas are used as references. (d-e) AFM thickness measurements in the drawn four regions. The circular foldings are related to the imprinted deformation that arises due to the pre-patterned cavities where the graphene was conformally grown.

S4. Electrostatic force and displacement (FEA)

The FEA pull-in voltages, solutions can not converge for $V_{bias} > 3.3$ V (geom. A), 3.8 V (geom. C), with respective max downward deformation of 1.45 µm and 1.10 µm. A static displacement of 1.65 µm, that is ≈ 1/3 of the gap (also considered as pull-in unstable position) is found at 8 V for geom. B.

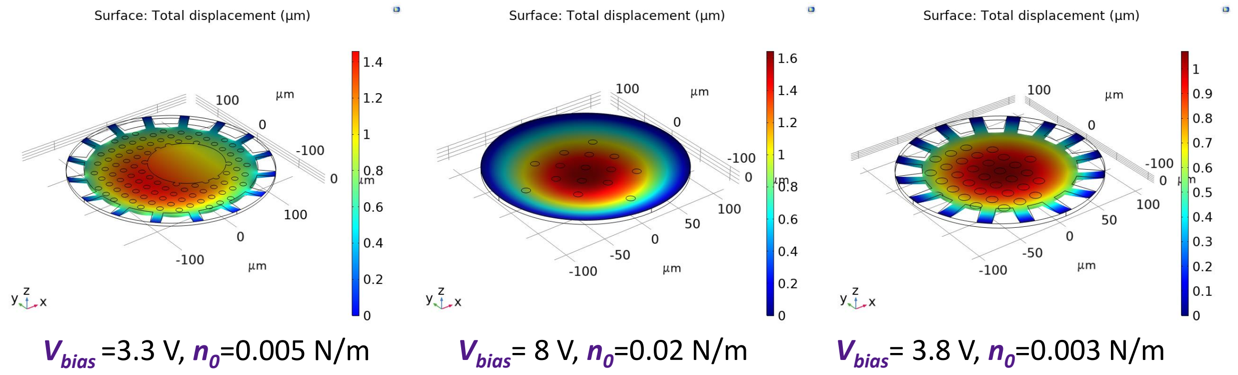

Figure S4: **FEA simulations (COMSOL Multiphysics).** The three geometries are modeled with different V_{bias} to extract the membrane displacement. Solutions geom. A, C couldn't converge at higher voltages of 3.3 V, 3.8 V without reaching the unstable point of 1/3 of the gap (pull-in region). Solution of geom. B reached a point of ≈ 1.6 µm ($1/3gap$) at 8 V.

S5. C-V characteristics and pull-in

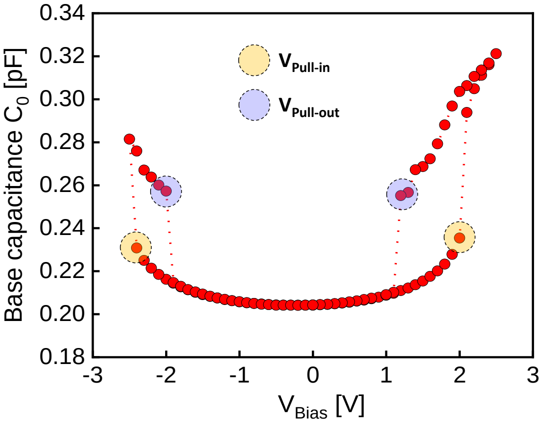

Figure S5: **Hysteresis behavior of C-V relation.** A DUT of geom. A is driven by the impedance analyzer with $V_{AC} = 100\,\mathrm{mV}$ at f_1=100 kHz and a V_{bias} sweep between -2.5 V - 2.5 V. The high potential and high current contacts are connected to the graphene membrane and the respective low terminals to the counter electrode. The hysteresis behavior is related to the non-linearity of the electrostatic force and is further enhanced by electrical charging of the residual TEOS layer in the back-plate and adhesive forces in the mechanical contact.

S6. Mechanical compliance measurement setup

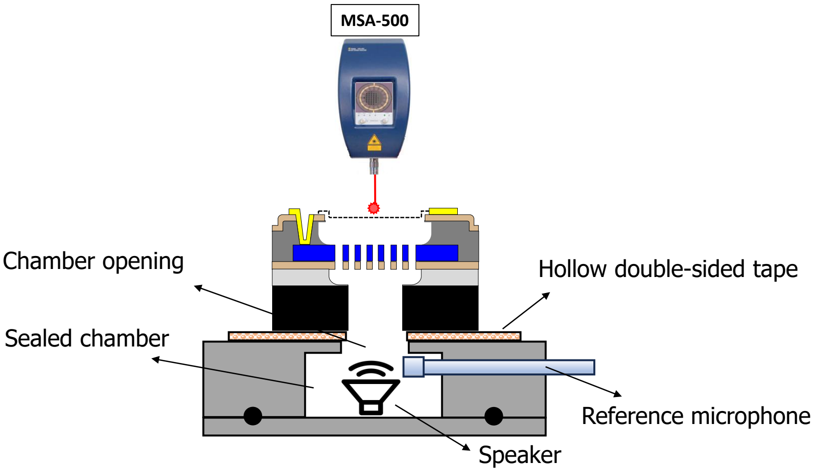

Figure S6: **Schematic setup for mechanical compliance measurements.** A Polytec MSA-500 is positioned above the diaphragm, focusing on its center, while the entire silicon chip is secured with a hollow double-sided tape in correspondence with the sealed chamber opening. Below this window, a reference microphone and a speaker are positioned. Both the speaker and reference microphones have been precisely calibrated to maintain a consistent sound pressure level of $94dB_{SPL}$ across the entire frequency range, ensuring a reliable seal.

S7. Damping analysis with lumped-element model

Despite the resonance frequencies $f_{01} > 20\,\mathrm{kHz}$ at low pressure (Figure 4), all devices exhibit cut-off frequencies within the audible range under sound actuation. In Figure S7a, a direct comparison between the experimental results and the simulated mechanical compliances at 94 $\mathrm{dB_{SPL}}$ is presented. With the incorporation of an optimized back-plate ($R_{BP} \approx 1 - 8 \cdot 10^8[\frac{Pa \cdot s}{m^3}]$) , all proposed devices exhibit a flat response up to $f_{01} = 16\,\mathrm{kHz}$ (Geom. A), 63kHz (Geom. B), and 25.1kHz (Geom. C) (Figure S7a - dashed lines). These simulated resonance frequencies remain lower than those obtained at low pressure, as discussed in the main manuscript (Figure 4), due to the impact of air-loading on the membrane.[1]

The low-pass behavior of the experimental results is then compared with the simulated

responses under high damping with a not-optimized back-plate (continuous lines), providing valuable insights into the damping magnitude of the proposed geometries. Considering the analytical acoustic resistance in Table 1 as $R_{BP} = 6.4 \cdot 10^{9}[\frac{Pa \cdot s}{m^3}]$ (Geom. A), $R_{BP} = 3.4 \cdot 10^{10}[\frac{Pa \cdot s}{m^3}]$ (Geom. B), and $R_{BP} = 1.35 \cdot 10^{10}[\frac{Pa \cdot s}{m^3}]$ (Geom. C), the model's findings indicate cut-off frequencies of 700 Hz (Geom. A), 3 kHz (Geom. B), and 950 Hz (Geom. C). However, these frequencies differ from the experimental values illustrated in Figure 6, which are 1500 Hz (Geom. A), 940 Hz (Geom. B), and 620 Hz (Geom. C).

In the case of Geom. A, an higher resonance in the experimental result is expected, possibly due to the larger back-plate opening caused by over-etching, which is not considered in the current analytical calculations for simplicity. On the other hand, the analytical calculations for Geom. B and C are likely overestimated owing to the considered assumption of equivalent hexagonal cell distributions for the venting openings covering the entire plate area as reported in previous works.[2] The current assumption is different for the proposed back-plates where the perforations are not distributed for the entire plate area. Consequently, the increased damping of the experimental results indicates an expected decrease in the resonance frequency. Moreover, any unopened venting holes are also not taken into account in the analytical calculations, resulting in a further increase in the resonance frequency for the analytical results.

The proposed results depicted in Figure S7a are simulated using a lumped model in LTSpice. Following the approach described in Beranek et al.[3] and other works concerning silicon-based MEMS microphones,[2,4] each component of the microphone can be represented as a lumped element. In this simplified model (Figure S7b), the first block signifies the voltage source, representing the magnitude of the pressure wave $p = 94dB_{SPL}$. The second block, represented by a resistor, characterizes the damping effect as F/v including all energy dissipation associated with the membrane's movements, primarily influenced by the viscous losses within the membrane back-plate system. The model essentially identifies four distinct regions that refer to a single hexagonal cell (A_{cell}): a squeeze film damping region existing between the

membrane and the back-plate (d_S), a segment accounting for the end effects of the viscous air flow within the channel (d_E), and an intermediate region connecting the squeeze-film (d_I) and a channel region formed by the perforation hole (d_C).[2] These four regions lead to the analytical damping factor as $d = d_S + d_E + d_I + d_C$, which is used to simulate the proposed devices. The damping factor refers to the single hexagonal cell A_{cell} that also includes the single perforation area A_{perf}. The third block characterizes the diaphragm, where the capacitance $C_{graphene}$ and the inductor $M_{graphene}$ correspond to the spring constant and mass of the proposed diaphragm. The fourth block accounts for the air-loading effect,[3] attributed to the higher gas density in the atmospheric environment where the microphone operates. In this scenario, $L1_{rad}$ denotes the air mass loading on the membrane while the components $R1_{rad}$, $R2_{rad}$, and $C1_{rad}$ allow accounting for frequency-dependent acoustic.[3]

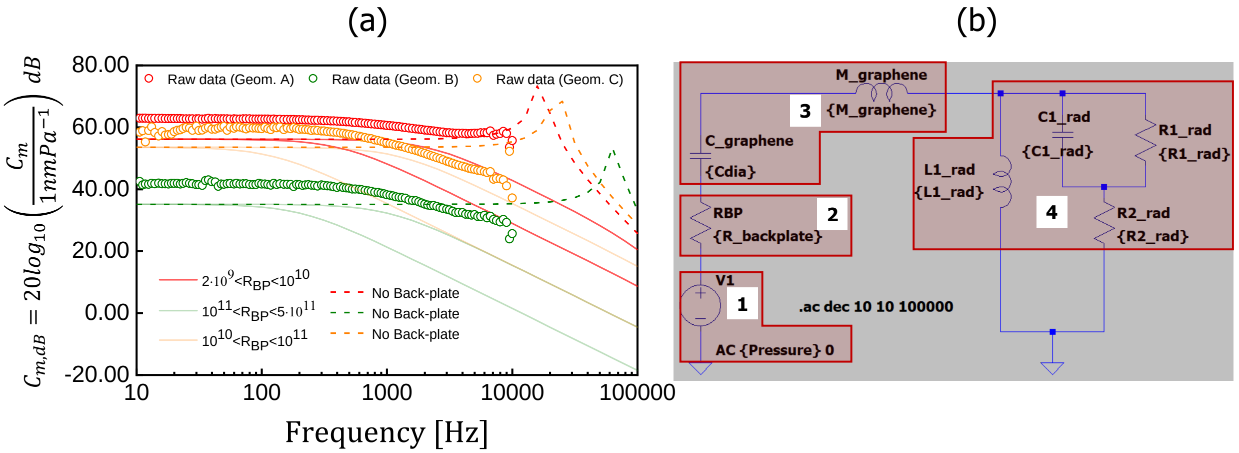

Figure S7: **Damping estimation of Geom. A, B, C based on lumped-element model.** (a) A comparison of experimental and simulated mechanical compliance is shown. The experimental results are denoted by empty circles, while the modeled results, considering very low damping with $R_{BP} \approx 1 \cdot 10^{-8}[\frac{Pa \cdot s}{m^3}]$ are represented by dashed lines. Notably, the damping magnitudes reported in the Figure S7 an inset are used to replicate the low-pass behavior observed in the experimental results. These inset R_{BP} intervals refer to the plotted semi-transparent line areas. The interval of values closely aligns with the analytical R_{BP}, derived using the prescribed equation and parameters from the geometries outlined in Table 1. (b) The figure includes a schematic of the lumped-element model employed to generate the modeled responses, utilizing a network similar to LTSpice.

Table 1: Parameter calculations for lumped modeling of Geom. A, B, C.
($\psi = A_{perf}/A_{cell}$, $A_{eff} = \frac{1}{3}\pi R^2_{graphene}$)

**Element**	**Geom. A**	**Geom. B**	**Geom. C**
$d_S = \frac{3\eta A^2_{cell}(4\psi - \psi^2 - 3 - 4\ln(\sqrt{\psi}))}{2\pi gap^3}\ [\frac{N\cdot s}{m}]$	$1.16\cdot10^{-8}$	$8.82\cdot10^{-8}$	$1.16\cdot10^{-8}$
$d_E = \frac{3\eta A_{cell}A_{plate}}{2R^3_{perf}}\ [\frac{N\cdot s}{m}]$	$2.88\cdot10^{-8}$	$1.23\cdot10^{-7}$	$2.88\cdot10^{-8}$
$d_I = \frac{3\eta 0.84 A^2_{plate}}{2R_{perf}gap^2}\ [\frac{N\cdot s}{m}]$	$1.95\cdot10^{-8}$	$9.38\cdot10^{-8}$	$1.95\cdot10^{-8}$
$d_C = \frac{8\eta t_{BP}A_{cell}A_{plate}}{\pi R^4_{perf}}\ [\frac{N\cdot s}{m}]$	$9.8\cdot10^{-9}$	$4.20\cdot10^{-8}$	$9.8\cdot10^{-9}$
$C_{graphene} = \frac{R^4_{graphene}\pi}{12n_0}\ [\frac{m^3}{Pa}]$	$3.65\cdot10^{-14}$	$1.53\cdot10^{-15}$	$1.27\cdot10^{-14}$
$M_{graphene} = \frac{9\rho_{graphene}t_{graphene}}{5R^2_{graphene}\pi}\ [\frac{kg}{m^4}]$	355.3468	751.4270	751.4270
$L1_{rad} = \frac{0.27\rho_{air}}{R_{graphene}}\ [\frac{kg}{m^4}]$	$2.0328\cdot10^{3}$	$2.95\cdot10^{3}$	$2.95\cdot10^{3}$
$C1_{rad} = \frac{5.94R^3_{graphene}}{\rho_{air}c^2}\ [\frac{m^3}{Pa}]$	$1.715\cdot10^{-16}$	$5.58\cdot10^{-17}$	$5.58\cdot10^{-17}$
$R1_{rad} = \frac{0.1404\rho_{air}c}{R^2_{graphene}}\ [\frac{Pa\cdot s}{m^3}]$	$2.26\cdot10^{9}$	$4.79\cdot10^{9}$	$4.79\cdot10^{9}$
$R2_{rad} = \frac{0.318\rho_{air}c}{R^2_{graphene}}\ [\frac{Pa\cdot s}{m^3}]$	$5.12\cdot10^{9}$	$1.08\cdot10^{10}$	$1.08\cdot10^{10}$
$R_{BP} = \frac{d_S+d_E+d_I+d_C}{A_{eff}Acell}\ [\frac{Pa\cdot s}{m^3}]$	$6.4\cdot10^{9}$	$3.45\cdot10^{10}$	$1.35\cdot10^{10}$

References

(1) Pezone, R. Air-loading effect on sub-mm polymer-free graphene membranes for microphone applications. **In prep. 2023**,

(2) Anzinger, S.; Wasisto, H. S.; Basavanna, A.; Fueldner, M.; Dehé, A. Non-Linear Behavioral Modeling of Capacitive MEMS Microphones. 2023 IEEE 36th International Conference on Micro Electro Mechanical Systems (MEMS). 2023.

(3) Beranek, L.; Mellow, T. *Acoustics: Sound Fields, Transducers and Vibration*; Elsevier, 2019; pp 143–229.

(4) Veijola, T. Analytic Damping Model for an MEM Perforation Cell. *Microfluidics and Nanofluidics* **2006**, *2*, 249–260.
