# Supplementary figures and images for: Highly-sensitive wafer-scale transfer-free graphene MEMS condenser microphones
Source: Microsyst Nanoeng. 2024 Feb 21;10:27. doi: 10.1038/s41378-024-00656-x (PMC10879197; doi:10.1038/s41378-024-00656-x)

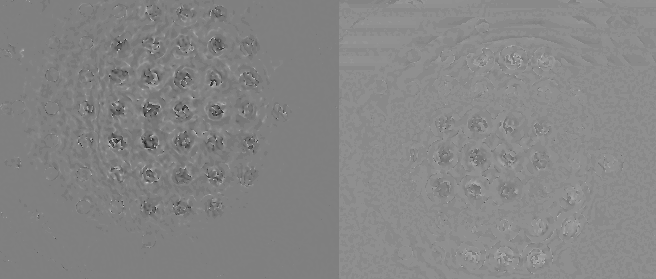

Supplement: Supplementary file 2 — Movie 1: LyncéeTec membrane modes (Geom.C) [file 41378_2024_656_MOESM2_ESM.gif]
